# Phased Diploid Genome Sequence for the Fast-Growing Microalga *Picochlorum celeri*

**DOI:** 10.1128/MRA.00087-20

**Published:** 2020-05-14

**Authors:** Scott A. Becker, Roberto Spreafico, Jennie L. Kit, Rob Brown, Maria Likhogrud, Wei Fang, Matthew C. Posewitz, Joseph C. Weissman, Randor Radakovits

**Affiliations:** a Synthetic Genomics, Inc., La Jolla, California, USA; b ExxonMobil Research and Engineering Company, Annandale, New Jersey, USA; c Department of Chemistry and Geochemistry, Colorado School of Mines, Golden, Colorado, USA; Vanderbilt University

## Abstract

Picochlorum celeri is a fast-growing marine microalga with high biomass productivity. Here, we report the use of PacBio sequencing to assemble the phased diploid genome of *P. celeri*.

## ANNOUNCEMENT

Picochlorum celeri (*Chlorophyceae*) is an algal species that is of commercial interest due to its high photoautotrophic reproductive rates and biomass productivity (1). Various *Picochlorum* species have been studied for potential application in biomass production (1–5), aquaculture feedstock (6, 7), and wastewater remediation (8). In recent years, several *Picochlorum* genome assemblies have been published (2, 9–11) and some of these are proposed to be diploid (10). Here, we report the first fully phased diploid *Picochlorum* genome assembly published to date (the organism has two copies of each chromosome; we represent the linked differences between them consistently along each scaffold).

*P. celeri* was isolated from the Gulf Coast of Texas in June 2015 and grown in enriched Instant Ocean seawater medium (1). For PacBio and Illumina sequencing, cell lysis of duplicate biological samples was accomplished through bead beating for 3 min in a Mini-BeadBeater (Biospec Products, Inc.) with 1-mm beads from OPS Diagnostics (PFAW 1000-100-21). Following lysis, total DNA was extracted using the Qiagen DNeasy PowerPlant Pro kit according to the manufacturer’s instructions. PacBio libraries were made using the SMRTbell template preparation kit with a molecular size cutoff of 10 kb. Illumina libraries were prepared using the TruSeq DNA LT sample preparation kit with a standard molecular size of 350 bp.

We obtained long reads from one single-molecule real-time (SMRT) cell on the Sequel instrument (Pacific Biosciences, Menlo Park, CA, USA) and short reads from the Illumina NextSeq system. The short reads were subsampled to 100× coverage and used only with GenomeScope (version 1.0.0) (12) for ploidy estimation; two kmer lengths (21 and 27) and two samples of reads gave heterozygosity estimates between 0.91% and 0.96%, indicating a diploid genome. The long reads were assembled with FALCON-Unzip (version 1.1.4, included in the pb-assembly conda recipe downloaded in December 2018) (13). In all, we gathered 273,487 long reads, with a mean insert length of 6,407 bp and an *N*_50_ of 9,250 bp. We worked with Phase Genomics (Seattle, WA, USA) to prepare a Hi-C library using a Phase Genomics Proximo Hi-C Plant kit, which is a commercially available version of the Hi-C protocol (14). Following the manufacturer’s instructions for the kit, intact cells from two samples were cross-linked using a formaldehyde solution, digested using the Sau3AI restriction enzyme, and proximity ligated with biotinylated nucleotides to create chimeric molecules composed of fragments from different regions of the genome that were physically proximal *in vivo* but not necessarily genomically proximal. Continuing with the manufacturer’s protocol, molecules were precipitated with streptavidin beads and processed into an Illumina-compatible sequencing library. Quality control for the library was performed by sequencing a small number of read pairs (556,109 read pairs) on an Illumina iSeq system and then aligning the reads using BWA-MEM (version 0.7.17) (15) with the -5SP and -t 8 options specified. The alignment was assessed for true Hi-C pairs in which forward and reverse reads were not found genetically proximal. Notably, the percentage of high-quality read pairs that aligned >10 kb apart on contigs longer than 10 kb was 13.93% (expected, 1 to 15%), the percentage of intercontig high-quality read pairs on contigs longer than 10 kb was 35.79% (expected, 10 to 60%), and the percentage of same-strand high-quality read pairs was 9.78% (expected, 2 to 50%). Sequencing was performed on an Illumina HiSeq 4000 system, generating a total of 164,537,658 PE150 read pairs.

FALCON-Phase (version 2) (16) was run using default parameters to correct likely phase-switching errors in the primary contigs and alternate haplotigs from FALCON-Unzip and output its results in pseudohap format, creating one complete set of contigs for each phase. Hi-C reads were then aligned to phase 0 contigs following the manufacturer’s recommendations (17). Briefly, reads were aligned using BWA-MEM with the -5SP and -t 8 options specified (all other options, default). SAMBLASTER (version 0.1.24) (18) was used to flag PCR duplicates, which were later excluded from analysis. Alignments were then filtered with SAMtools (version 1.9) (19) using the -F 2304 filtering flag to remove nonprimary and secondary alignments.

The Phase Genomics Proximo (version hash d33cacdd) Hi-C genome scaffolding platform was used to create chromosome-scale scaffolds from the FALCON-Phase phase 0 assembly, following the same single-phase scaffolding procedure described by Bickhart et al. (20). As in the LACHESIS method (21), this process computes a contact frequency matrix from the aligned Hi-C read pairs, normalized to the number of Sau3AI restriction sites (GATC) on each contig, and constructs scaffolds in such a way as to optimize expected contact frequency and other statistical patterns in the Hi-C data. Approximately 120,000 separate Proximo runs were performed to optimize the number of scaffolds and scaffold construction in order to make the scaffolds as concordant with the observed Hi-C data as possible. This process resulted in a set of 5 preliminary chromosome-scale scaffolds containing 13.5 Mbp of sequence (98.5% of the input assembly).

Juicebox (version 1.9.8) (22, 23) was then used to correct scaffolding errors, resulting in a total of 12 chromosome-scale scaffolds. FALCON-Phase was run a second time to detect and correct phase-switching errors that were not detectable at the contig level but were detectable at the chromosome-scale scaffold level. Metadata generated by FALCON-Phase for scaffold phasing were used to generate matching .assembly files (a file format used by Juicebox) for each phase and subsequently used to produce a diploid, fully phased, chromosome-scale set of scaffolds using a purpose-built script (https://github.com/phasegenomics/juicebox_scripts).

We polished the diploid assembly twice sequentially with the long reads, aligning with pbmm2 (version 1.0.0) and polishing with Arrow (version 2.3.3) (both available at https://github.com/PacificBiosciences/pbbioconda). Long-read polishing was stopped after two rounds when the consensus quality was estimated to be better than Q40 (a third round with Arrow suggested fewer than 1 in 10,000 changes). Short-read polishing was not done due to the risk of incorrectly merging the two phases of the genome (Shawn Sullivan, Phase Genomics, personal communication). The final phased and scaffolded genome consists of two phases totaling 27.43 Mbp spread across two pairs of 15 scaffolds each, with a scaffold *N*_50_ of 1.151 Mbp and an overall G+C content of 46%. The two phases of the genome were aligned and analyzed with the nucmer and dnadiff programs from the MUMmer4 suite (version 4.0.0.beta2) (24), finding a total of 112,875 single-nucleotide polymorphisms (SNPs) between the two phases (SNP heterozygosity, 0.8%). Assemblytics software (version available on 15 August 2016, git commit hash c937e96d) (25) was used to analyze the structural variation of the two phases of the genome and found 16,794 indels and larger structural variants affecting 558.74 kb. Default parameters were used for all software unless otherwise specified. This phased assembly will enable future studies to better understand the photosynthetic efficiency of *P. celeri*.

### Data availability.

This whole-genome shotgun project has been deposited at DDBJ/ENA/GenBank under the accession number JAACMV000000000. The raw data are available under the accession number PRJNA598876.
